# The 15-year national trends of urinary cancers incidence among Iranian men and women; 2005–2020

**DOI:** 10.1186/s12939-023-02084-1

**Published:** 2024-01-22

**Authors:** Amir-Hossein Mousavian, Gita Shafiee, Ali Sheidaei, Narges Zargar Balajam, Mehdi Ebrahimi, Fatemeh Khatami, Kimiya Gohari, Alisam Aryan, Ali Ghanbari-Motlagh, Afshin Ostovar, Seyed Mohammad Kazem Aghamir, Ramin Heshmat

**Affiliations:** 1 Chronic Diseases Research Center, Endocrinology and Metabolism Population Sciences Institute, Tehran University of Medical Sciences, Tehran, Iran; 2 Department of Epidemiology and Biostatics, School of Public Health, Tehran University of Medical Sciences, Tehran, Iran; 3 Department of Internal Medicine, Faculty of Medicine, Sina Hospital, Tehran University of Medical Sciences, Tehran, Iran; 4 Urology Research Center, Tehran University of Medical Sciences, Tehran, Iran; 5 Department of Biostatistics, Faculty of Medicine Sciences, Tarbiat Modares University, Tehran, Iran; 6 Department of Radiotherapy, School of Medicine, Shahid Beheshti University of Medical Sciences, Tehran, Iran; 7 Osteoporosis Research Center, Endocrinology and Metabolism Clinical Sciences Institute, Tehran University of Medical Sciences, Tehran, Iran

**Keywords:** Bladder cancer, Renal cancer, Urinary system, Epidemiology, Incidence

## Abstract

**Background:**

Urinary tract cancers including bladder, kidney, ureter, and pelvis are a common malignancy worldwide with high mortality ratio. Aimed to investigate the prevalence of these cancers, we conducted this study.

**Methods:**

In this study, all the information related to ICD10 codes, gender, age and province of residence of individuals were obtained from the data of Iran’s cancer registry by the Ministry of Health, Medicine and Medical Education and demographic evidence for each sub-country from the reports of Statistics Center of Iran (SCI). Also, the data of two Iranian national survey studies CASPIAN-III, IV, and V (information related to the care and prevention of non-communicable diseases (NCD) in childhood and adolescence) and STEPs (including information on NCD in adults over 18 years old) were used. The data was analyzed using Poisson regression with mixed effects to estimate the incidence of cancers.

**Results:**

Bladder and kidney neoplasm are the most common cancers of the urinary system in Iran. The prevalence of bladder cancer has increased from 5.82 to 11.50 per 100,000 individuals. The increasing trend is growing faster in men compared with women. The incidence of kidney neoplasm has increased over the years (2.03 in 2005 vs. 7.02 in 2020 per 100,000). Having a higher incidence ratio compared with bladder cancer, kidney cancer is responsible for 35.06% of all urinary cancers in 2020 compared with 23.71% in 2005. Both neoplasms of the ureter and renal pelvis were recorded rarely and with lower incidence in both sexes during this period.

**Conclusion:**

Considering the increasing trend in the incidence of urinary neoplasms in Iran during these years, the advantage of focusing on the risk of urinary cancers is highlighted. Therefore, investigating the prevalence and incidence of urinary cancers to plan and manage these cancers will result in prevention and reduction of the disease burden on the Iranian society. Future studies in this field can help in the prevention and well-timed diagnosis of these cancers.

**Supplementary Information:**

The online version contains supplementary material available at 10.1186/s12939-023-02084-1.

## Introduction

Bladder malignancy is the 10th most common cancer of the urinary tract in people over 60 years old worldwide [1, 2]. Cancers of the urinary system encompass a group of diseases including small benign tumors to destructive malignant with high morbidity [3]. More than 90% of urinary tract malignancies are related to urothelial or transitional cell carcinomas, which comprise bladder and upper urinary tract (UUT) cancers (two-thirds of them in the renal pelvis and the rest in the ureter) [4, 5]. Also, male: female proportion ranging of bladder cancer is 2 to 6, so men are affected more than women by this cancer. The incidence of bladder cancer at the international level is correlated to the prevalence of smoking and occupational exposure [1, 6]. In a study by Brown KF et al. in the UK, smoking causes bladder cancer in 46.0% of men and 41.5% of women [7].

The renal pelvis tumors of the upper urinary tract are only 5% of all cases of urinary system malignancies and urothelial carcinoma in the ureter is even less [8]. The report of Siegel et al., in 2013, demonstrated a 2:1 ratio of urothelial carcinoma of the upper urinary tract in men compared to women [9].

In 2020, 573,278 new cases and 212,536 demises due to bladder tumor were reported globally. Moreover, 80,000 new cases and 17,000 deaths of bladder cancer are announced annually in the United States [10, 11]. An almost 80% improvement in five-year survival rates for bladder cancer has been reported in the United States since the 1970s. Additionally, the death rate related to urinary system cancers in Western European countries has been decreasing in the last 20 years, while it displays growth in some Eastern European countries [12, 13].

In a retrospective study on 2117 patients with urinary tract cancer in Isfahan, Iran, the incidence rate of these malignancies was 66.4% and 17.9% in men and women, respectively. This study also shows that the incidence rate of urinary system tumors increased during the years 2011 to 2015. Bladder cancer had the highest prevalence (33.2%) and ureter the lowest (0.36%), among urinary cancers. Also, the mortality rate related to these cancers is at 11% during this period [14]. Due to the importance of urinary system cancers and the impact of prevention and early detection strategies on decreasing the burden of these malignancies, it is necessary to investigate these malignancies’ incidence rates and epidemiological information to accomplish and create essential strategies. The purpose of this study was to evaluate the 15-year trend of urinary tract cancers from 2005 to 2020 in the Iranian people.

## Methods

### Data sources

In this research, information including ICD10 codes for malignant neoplasm type, sex, age, and the province of residence of individuals was extracted from the data of the Iranian cancer registry for the years 2008 to 2010, 2014, and 2015, which were collected by the Ministry of Health and Medical Education. This is a population-based registry with almost complete coverage and automatic data cleaning. Therefore, we did not have to calculate incompleteness, which is necessary for similar studies. However, the registry missed some demographic variables like age and sex. To overcome this challenge, the multiple imputation bootstrapping-based algorithm by Amelia package in R software was used to estimate the proportion of missing values of each variable (Age, Sex, and province all less than 5% missing) [15]. As the missing values were related to the errors in registration, not the attributes of the cancer type, the at least missing at random were supposed to be satisfied.

Several factors such as the length of groups, ideal cut points, the least valid age, and the way of meaning for the last group were considered in the definition of age groups. Therefore, according to the previous studies, the global burden of diseases (GBD) and conducting 5-fold cross-validation the age range of people was selected from 15 to + 75 years old, in groups with a length of 10 years [16, 17].

Demographic data based on age and gender groups for each sub-national division was obtained from the reports of the Statistics Center of Iran (SCI) for the population and housing census of 2001, 2006, 2011, and 2016 [18]. For the other years which did not hold a national census, the SCI published the expected frequency of population by age, sex, and province. It is possible to access this information online through the SCI website [19].

The non-possibility of examining the relationship between cancer registry information and covariates at the individual level led to the use of an ecological approach. Thus, people in a province with a common geographic feature and a similar age, and gender group formed the subject groups. As a result, this manner made it possible to use covariates from other data sources.

Urinary cancers are classified as non-communicable diseases. Hence, to build the prediction model, we focused on the available covariates in the surveys designed to study the risk factors of non-communicable diseases. In this regard, the data of two Iranian national survey studies of CASPIAN and STEPs were applied. Both of these studies follow the guidelines of the World Health Organization (WHO). There are guidelines for these studies, so the data gathering has been conducted strictly and systematically.

STEPs data is related to 6 survey steps (in 2005, 2007, 2008, 2009, 2011, and 2016) that focus on the risk factors of non-communicable diseases in adults over 18 years old [20]. The CASPIAN studies covered information on the care and prevention of non-communicable diseases of childhood and adolescence in the years CASPIAN-III (2009–2010), CASPIAN-IV (2011–2012), and CASPIAN-V (2015) [21, 22].

Using a population data set based on census information, the variable of urbanization ratio (urban residents to individuals dwelling in rural parts) was entered into the model as an indicator for the distinction between urban-rural lifestyles.

### Covariates

Initially, covariates were collected at the individual level and subsequently compiled into a dataset encompassing all combinations of age, year, gender and province. The non-parametric spline smoothing method was used to compute unavailable real data and a uniform cubic spline was calculated using this function in R statistical software with Hyman filter [23].

### Statistical analysis

Poisson regression with mixed effects was applied for data analysis and incidence rate approximation. In this manner, the quantity of new cases within every age and gender category for all provinces was considered as the response variable in all available years. Separate analyzes were accomplished for each malignant neoplasm type. By randomizing based on year, we established cohesion between incident cases across times, and by province randomizing eliminated unknown factors of changes in provinces. Finally, the population at risk was used to balance the model [24].

### Model building and validation

Distinct models were designed for each malignant neoplasm. Details are described elsewhere, in a previous study [25]. R statistical software was used for all statistical analyzes and graph creation. Models were compared with Bayesian information criterion (BIC) and Akaike’s information method (AIC). In order to assess the robustness and reliability of the model, the data set was initially partitioned into five randomly selected subsets. Subsequently, during each iteration, four of these subsets were utilized for model construction, while the remaining subset was reserved for outcome validation, following a 5-fold cross-validation methodology. The identical approach was employed to select the optimal age cohorts. The root (square error) was used to appraise the models.

### Ethical consideration

This study was approved by the ethical committee of Tehran University of Medical Sciences (IR.TUMS.VCR.REC.1398.218).

## Results

**Fig. 1:**
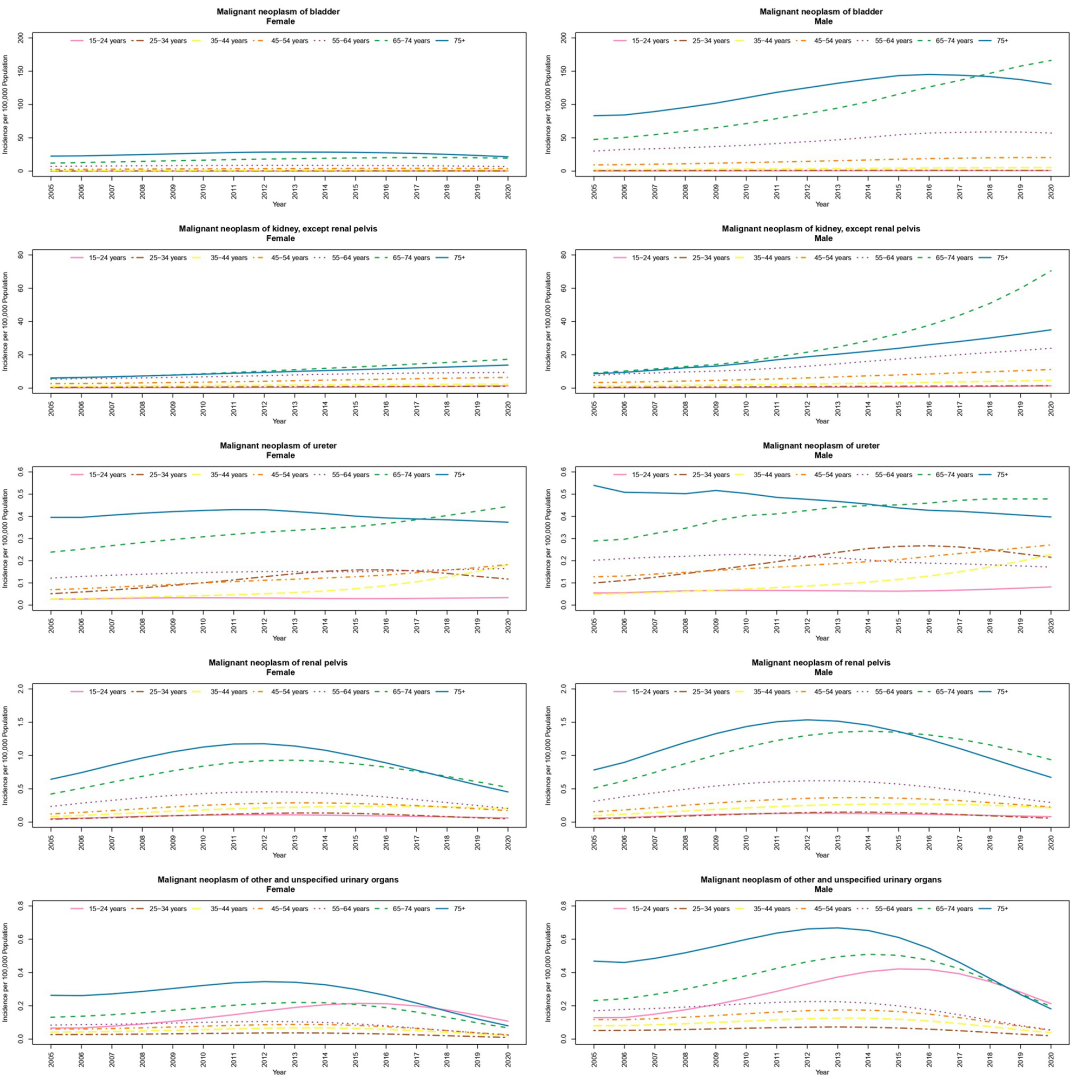
Age-Specific Trends of Incidence Rate of Malignant Neoplasms of urinary system in 100,000 Population

In 2005, malignant neoplasm of bladder has the highest incidence rate about 83.2 (82.89–83.52) and 22.52 (22.17–22.87) incidences per 100,000 for males and females of the 75 + years old age group, respectively. The incidence for the age group of 65 to 74 is 47.44 (47.28–47.61) per 100,000 for men and 11.96 (11.77–12.15) for women. These two groups have the most numbers of cancers recorded during the entire time of the study. The incidence for 75 + group is 130.65 (130.07-131.22) per 100,000 males and 21.48 (20.95–22.01) for females in 2020 respectively. There is an increasing pattern through the years in all age groups except 75 + group in females that has decreased in 2020 compared with 2015 (28.16 (27.99–28.33)) (Fig. 1). The incidence of malignant neoplasm of kidney in females is 6.21 per 100,000 in the 75 + age group and 5.81 for 65 to 74 age group, while in 2020 the incidence is more common among 65 to 74 age group compared with the 75 + years age group (17.31 vs. 13.82 per 100,000). As for the males, number of neoplasms recorded are significantly higher in 2020 (70.96 per 100,000) compared with 2005 (9.14 per 100,000) for 65 to 74 age group and the same pattern is detected in 75 + age group with 35.01 per 100,000 incidences in 2020 and 8.61 in 2005 respectively. Figure 1 demonstrates the changing trend of age-specific incidence rates from 2005 to 2020 for all types of urethral malignant neoplasms in both men and women. The crossed lines indicate the change in the age pattern of the incidence rate throughout the year. For example, the incidence rate of bladder malignancy in the age group over 75 years until 2017 and after this time was higher than the age group of 74 − 65 years.

**Fig. 2:**
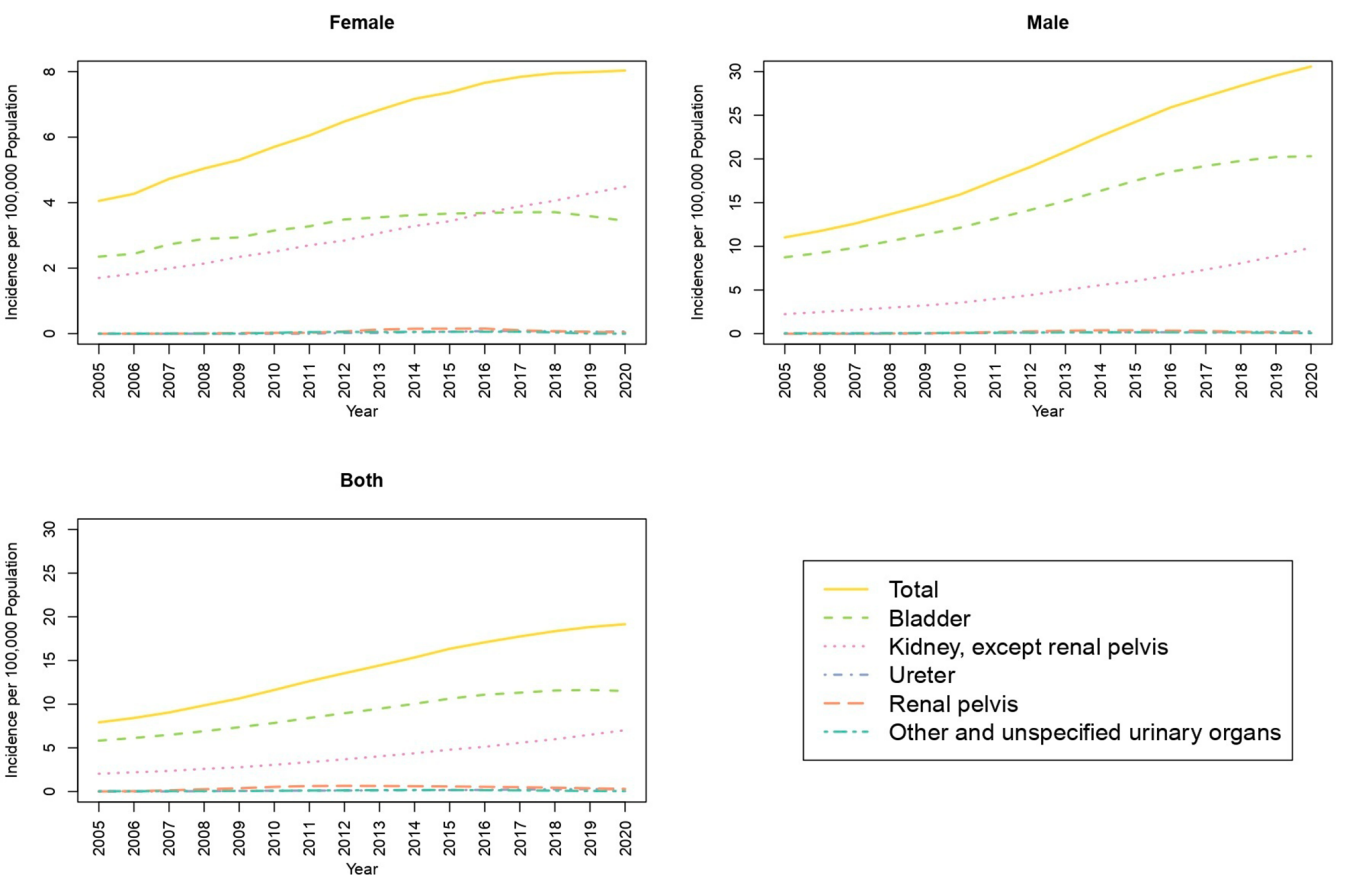
Age Standardized Incidence Rate of Malignant Neoplasms of urinary system in 100,000 population

Neoplasm of bladder has the uppermost age-standardized incidence in 2005 (5.82 per 100,000) in overall population and has nearly doubled in 2020 (11.50 per 100,000). This increase in mainly due to the increase of incidence in men (20.30 in 2020 compared with 8.74 per 100,000 in 2005). The second most occurring cancer in overall population is the neoplasm of kidney (7.02 per 100,000 in 2020). A significant increase of incidence in the neoplasm of kidney is detected over the years specially in women. Such that in 2020 the neoplasm of kidney has the most incidence with 4.48 per 100,000 women, surpassing neoplasm of bladder with 3.44 per 10,000 (Fig. 2). Both neoplasms of ureter and renal pelvis were recorded so rarely and had an incidence of less than 1 per 100,000 individuals in either sex throughout the study.

**Fig. 3:**
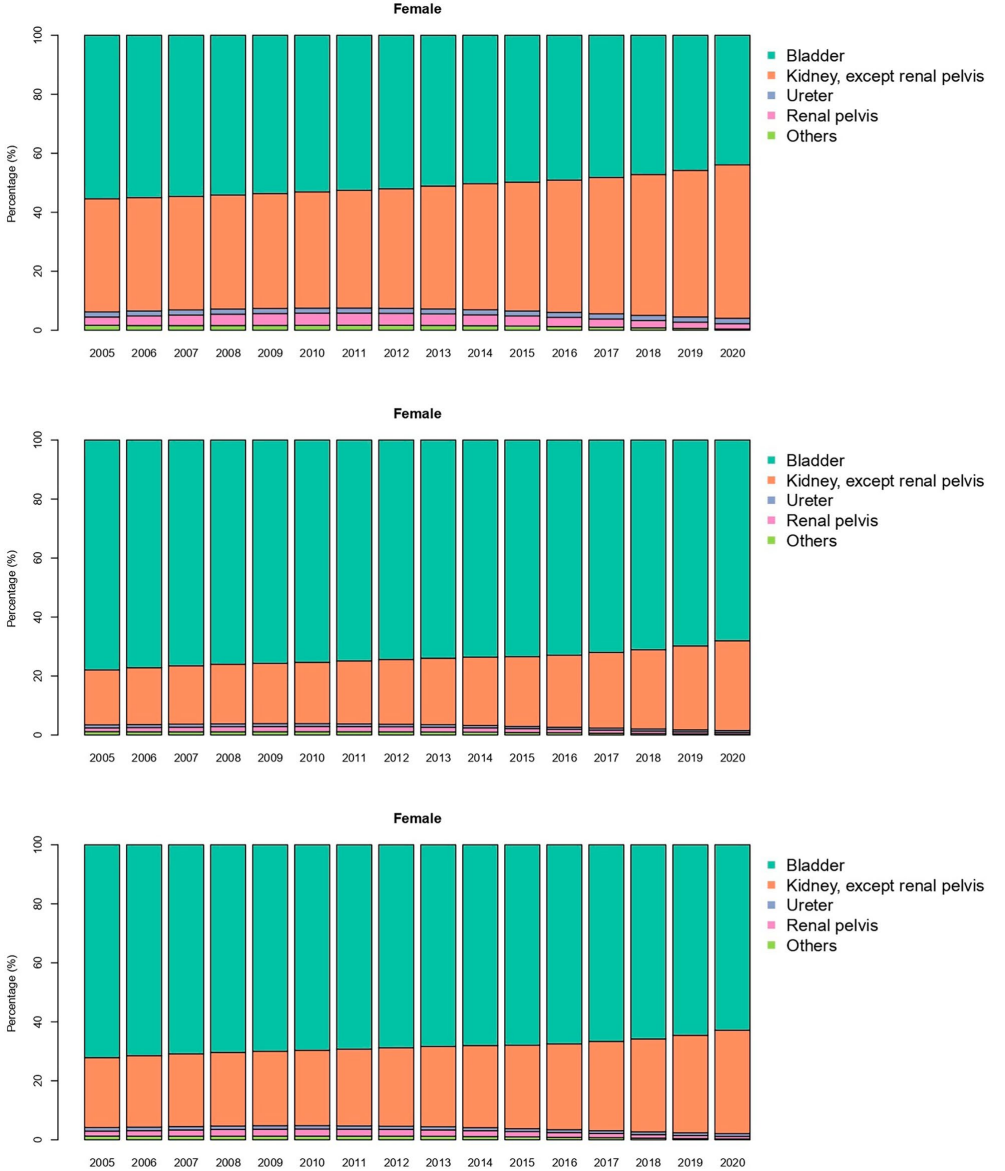
Percentage of each type of malignant neoplasms from the total Malignant Neoplasms of urinary system

Figure 3 shows the share (percentage) of each type of neoplasm in men, women and total population over the years. Neoplasm of bladder was responsible for 77.93% of all the detected urinary neoplasms in men in 2005 compared with 68.03% in 2020. Kidney neoplasms share has increased from 16.81 in 2005 to 30.42 in 2020. Ureter, renal pelvis, and other neoplasms each have a share of less than 1%. Percentage for neoplasm of bladder in female has dropped from 55.42 in 2005 to 43.94 in 2020, while neoplasm of kidney with 51.96% has taken neoplasm of bladder’s place as the most occurring ureteral neoplasm in women. Ureter and renal neoplasm percentage are 1.84 and 1.81 in 2020 respectively. In the overall population neoplasm of bladder is still the most common neoplasm detected although the percentage has dropped from 72.12 in 2005 to 62.85 in 2020. Kidney neoplasm is in the second place with 35.06% of all incidences recorded in 2020 compared with 23.71% recorded in 2005. Ureter, renal pelvis, and other neoplasms responsible for nearly 2% of all the urinary cancer incidence recorded in 2020 combined.

The supplementary Fig. 1 provides information on the geographical distribution of incidence rates across provinces in Iran for urinary neoplasms in the years 2005 and 2020. There was an increasing trend in the incidence rate in all provinces.

## Discussion

In this study, an estimation of national and subnational incidence rates, as well as trends for each type of urinary cancer in both genders, was conducted in Iran from 2005 to 2020. The overall quantity of identified cancers, regardless of sex, has increased over the duration of this study. A significant rise in the incidence of bladder cancer and kidney neoplasm is notable. However, the prevalence of renal pelvis, ureter, and other neoplasm have not change significantly and the patterns are the same.

The neoplasm of the bladder is following an increasing trend during 2005 to 2020 period. The prevalence is much higher in men but the number of neoplasms detected in younger age groups is greater in 2020 compared with 2005 in both genders. Since smoking and tobacco use are major risk factors for bladder cancer, this may explain why the prevalence of bladder cancer is higher among men [26, 27]. the increased number of smokers both in men and women and starting at a young age has resulted in higher number of bladder cancer in younger group ages compared with the past [28, 29]. It is mentionable that the effects of tobacco use appear several decades in the future. Bladder cancer is a global concern with approximately 550,000 new cases annually, it ranks as the ninth most frequently-diagnosed cancer worldwide as well as the sixth most prevalent malignancy in the United States [11, 30, 31]. The prevailing clinical manifestation is that of asymptomatic hematuria. Similar to our study, incidence rates are consistently lower in women than in men [30]. The population attributable risk (PAR) of tobacco smoking for bladder cancer is estimated at around 50% [32]. Therefore, a decrease in tobacco smoking prevalence will significantly diminish the incidence of bladder cancer, of course with a delay of several decades [11]. While smoking and tobacco are considered as main cause for bladder cancer, urinary tract infections are responsible, Schistosoma haematobium infection is known as the second leading cause for bladder cancer globally [33, 34]. Although Schistosoma haematobium infection is most detected in Africa and Middle East, but the infection is currently eradicated in Iran [35].

Since there is no recommendation for screening asymptomatic adults for bladder cancer, hence, increasing patient awareness combined with some policy actions such as high taxes on all tobacco products and prohibition of advertising might help with reducing the prevalence of tobacco use and bladder cancer eventually [31, 36].

Kidney cancer comprises of renal cell carcinoma (RCC), the predominant form, and renal transitional cell carcinoma (RTCC). The prevalence of kidney cancer has augmented over the past 15 years is Iran in both men and women. our results show that kidney neoplasm is currently the most detected neoplasm of ureteral cancers in females, replacing bladder cancer. The incidence of kidney cancer rises throughout the world but most notably in the United States and developed European countries [37–40]. This indicates the effect of advanced diagnostic imaging not to forget the impact of several risk factors such as obesity, smoking and hypertension [37]. Men, especially black men, are more likely to be affected than women [39]. A noteworthy positive association has been ascertained between the standardized incidence and mortality rate of kidney malignancy and the Human Development Index (HDI) [41]. Hematuria is a warning sign that dictates further evaluation and imaging [39]. Preventive actions for the risk of kidney cancers are a healthy diet and avoiding smoking according to the evidence [37].

According to our data, the prevalence of ureter and renal pelvis neoplasm are stable over the years and are responsible for less than 1% of ureteral cancers. Few studies have reported the prevalence of these neoplasms [42]. there is an association between occupational exposures/risk factors and risk of the renal pelvis malignancy. There is a possible association between exposure to asbestos and heavy metals [43]. Since asbestos is no longer used in buildings and construction, the stabilized prevalence of renal pelvis cancer seems justified. Future studies to detect the prevalence of these cancers and definite risk factors are required globally.

The study’s notable attribute lies in the extensive duration of data collection, which enabled a comprehensive analysis of the incidence trend for each neoplasm across both age-standardized rate and age-specific rate groups. Creating age groups with each group representing a decade and the starting group as early as 15 years old. Furthermore, the collection of data for each individual province has facilitated the identification of the areas with the highest number of occurrences. Healthcare systems and policymakers will benefit from this as to where and for whom to use the resources that will help the most. Several limitations were encountered during the course of this study, primarily due to the utilization of statistical modeling for data synthesis, which resulted in incomplete data within our registry. Also, the pathologic type of kidney cancers in this study is not explored separately. To our knowledge, no other investigation has demonstrated the prevalence of these malignances in Iran.

## Conclusions

A study we conducted to observe the changing trend for each ureteral neoplasm for a duration of 15 years in both males and females. Our study encompassed data pertaining to each age group along with trends in cancer incidence in every province of Iran over 15 years. Bladder and kidney cancers are the most occurring cancers during our study. The growing use of tobacco has resulted in greater numbers of bladder cancer at younger ages. Increased prevalence of obesity and hypertension has resulted in more kidney cancers, especially in women. The prevalence of renal pelvis and ureter cancers has been low and stable over the years.

### Electronic supplementary material

Below is the link to the electronic supplementary material.

**Supplementary Material 1: Fig. 1.** Geographical distribution of Malignant Neoplasms of urinary system incidence rates per 100,000 population in 2005 and 2020

**Supplementary Material 2: Fig. 2.** Map of Iran included indications of provinces

## Data Availability

We would like to inform all the reviewers that our data, analytic methods, and study materials are available upon request, by contacting our corresponding author.
