# Supplementary figures and images for: The 15-year national trends of urinary cancers incidence among Iranian men and women; 2005–2020
Source: Int J Equity Health. 2024 Jan 22;23:13. doi: 10.1186/s12939-023-02084-1 (PMC10804628; doi:10.1186/s12939-023-02084-1)

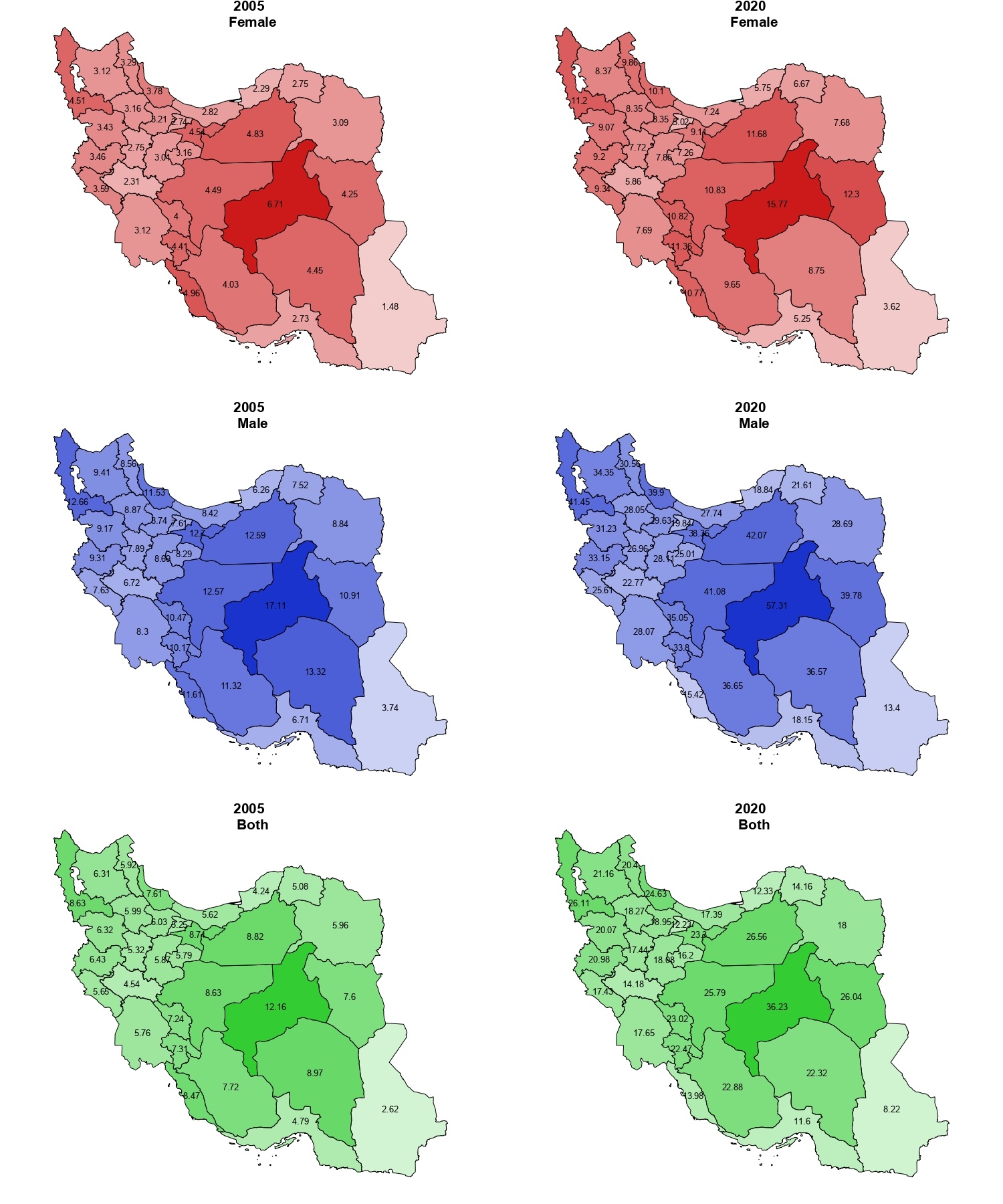

Supplement: Supplementary file 1 — Supplementary Material 1: Fig. 1. Geographical distribution of Malignant Neoplasms of urinary system incidence rates per 100,000 population in 2005 and 2020 [file 12939_2023_2084_MOESM1_ESM.jpg]

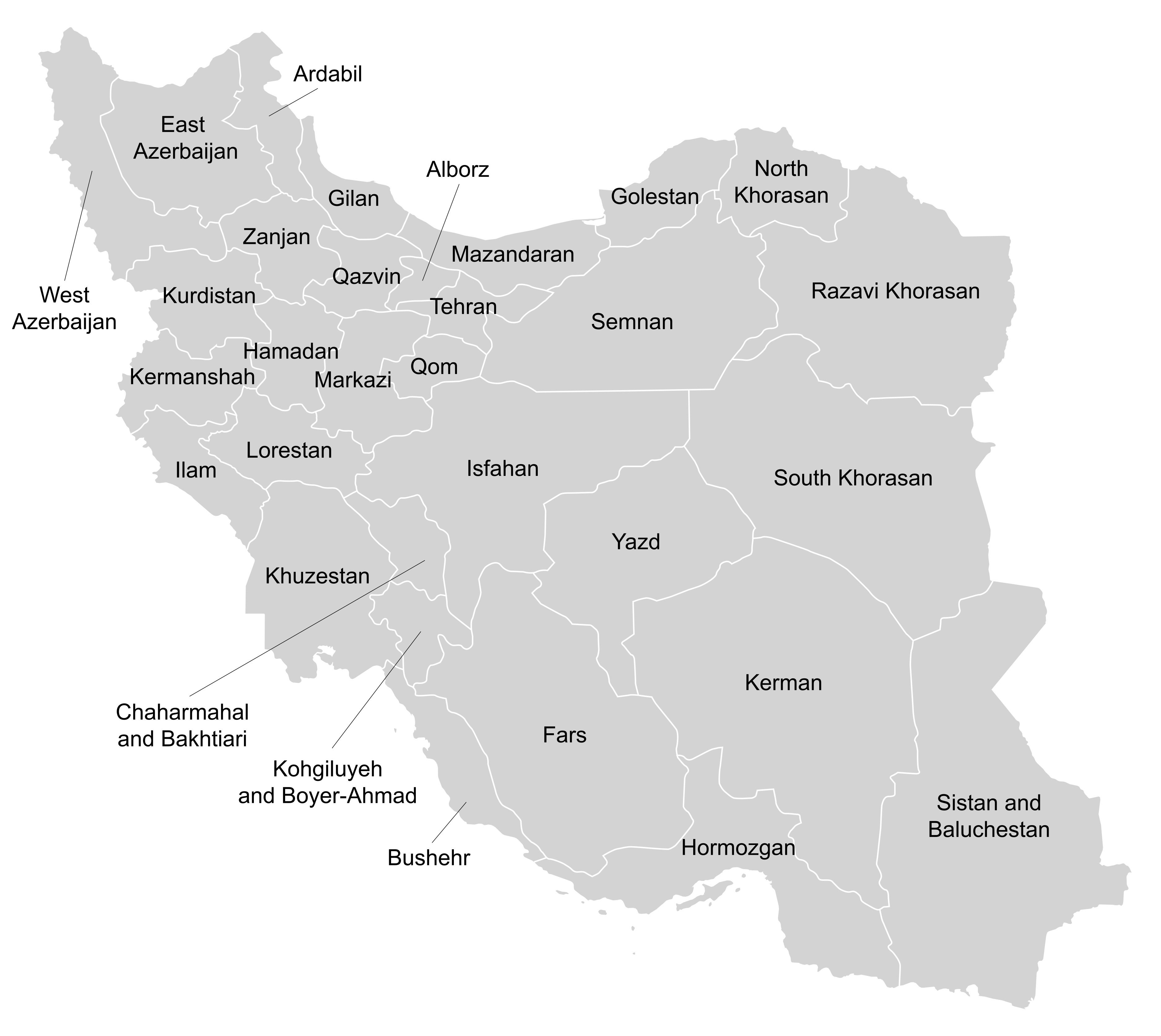

Supplement: Supplementary file 2 — Supplementary Material 2: Fig. 2. Map of Iran included indications of provinces [file 12939_2023_2084_MOESM2_ESM.jpg]
